# Expert Consensus on the Diagnosis and Treatment of Anticancer Drug-Induced Interstitial Lung Disease

**DOI:** 10.1007/s11596-022-2693-2

**Published:** 2023-03-03

**Authors:** Hua-ping Dai, Fei Ma, Yan-hong Ren, Shan-shan Chen, Yi-qun Li

**Affiliations:** 1 Department of Pulmonary and Critical Care Medicine, China-Japan Friendship Hospital, National Center for Respiratory Medicine, Chinese Academy of Medical Sciences, Beijing, 100029 China; 2 Department of Medical Oncology, National Cancer Center, National Clinical Research Center for Cancer, Cancer Hospital, Chinese Academy of Medical Sciences and Peking Union Medical College, Beijing, 10021 China

**Keywords:** drug-induced interstitial lung disease, anticancer drug, diagnosis, treatment

## Abstract

Drug-induced interstitial lung disease (DILD) is the most common pulmonary adverse event of anticancer drugs. In recent years, the incidence of anticancer DILD has gradually increased with the rapid development of novel anticancer agents. Due to the diverse clinical manifestations and the lack of specific diagnostic criteria, DILD is difficult to diagnose and may even become fatal if not treated properly. Herein, a multidisciplinary group of experts from oncology, respiratory, imaging, pharmacology, pathology, and radiology departments in China has reached the “expert consensus on the diagnosis and treatment of anticancer DILD” after several rounds of a comprehensive investigation. This consensus aims to improve the awareness of clinicians and provide recommendations for the early screening, diagnosis, and treatment of anticancer DILD. This consensus also emphasizes the importance of multidisciplinary collaboration while managing DILD.

Drug-induced lung injury is an injury in the respiratory system, i.e., the airways, lung parenchyma, pulmonary vessels, and pleura, that results from drug administration. The most common drug-induced lung injury is drug-induced interstitial lung disease (DILD)[1, 2]. In recent years, with the emergence of new anticancer agents such as tyrosine kinase inhibitor (TKI), mammalian target of rapamycin (mTOR), antibody-drug conjugates (ADCs), and immune checkpoint inhibitors (ICIs), the incidence of anticancer DILD has gradually increased[1]. However, compared with drug-induced liver and kidney injuries, drug-induced lung injury has not attracted significant attention in clinical practice[3].

Misdiagnoses and missed diagnoses of DILD are not rare due to the diverse clinical manifestations and the lack of specific diagnostic methods[2]. If not treated properly, it may interrupt or discontinue anticancer treatment or even become life-threatening in severe cases. As a result, it is very important for clinicians to promptly recognize, identify, and manage anticancer drug-induced lung injury. Herein, we discussed the diagnosis and treatment of DILD with experts from the respiratory, oncology, imaging, pathology, and pharmacology, and radiology departments and finally reached a consensus on the management of anticancer DILD in China.

## 1 EPIDEMIOLOGY

In 1880, Osler, a Canadian physician, found during an autopsy that excessive heroin may cause acute pulmonary edema and reported the first drug-induced lung injury[4]. In 1972, Rosenow systematically described the relationship between more than 20 drugs and lung injury, and drug-induced lung injury has been

Corresponding authors, Fei MA, E-mail: drmafei@126.com, Department of Medical Oncology, National Cancer Center, National Clinical Research Center for Cancer, Cancer Hospital, Chinese Academy of Medical Sciences and Peking Union Medical College, Beijing 10021, China; Hua-ping DAI, E-mail: daihuaping@ccmu.edu.cn, Department of Pulmonary and Critical Care Medicine, China-Japan Friendship Hospital, National Center for Respiratory Medicine, Chinese Academy of Medical Sciences, Beijing 100029, China
*This work was supported by grants from CAMS Innovation Fund for Medical Sciences (CIFMS) (No. 2021-I2M-1-014) and National Key R & D Program of China (No. 2021YFC2500700).
Fei MA and Hua-ping DAI, on behalf of the Anticancer Drug-induced Interstitial Lung Disease Diagnosis and Treatment Group. The Chinese version of the article entitled "Expert Consensus on the Diagnosis and Treatment of Anticancer Drug-Induced Interstitial Lung Disease" was initially published in National Medical Journal of China (Chinese): 2022,44(7):693-702. Doi: 10.3760/cma.j.cn112152-20220412-00244

gradually recognized and reported[5]. So far, over 1300 drugs have been reportedly associated with pulmonary toxicity[1]. Among them, anticancer drugs are the most common agents that could cause interstitial lung disease. The incidences of ILD induced by different anticancer drugs are summarized in table 1. Chemotherapeutic agents (bleomycin, cyclophosphamide, methotrexate), tyrosine kinase inhibitors (gefitinib, erlotinib, afatinib), mTOR inhibitors (everolimus, sirolimus), ICIs (pembrolizumab, atezolizumab) and ADCs (trastuzumab emtansine, trastuzumab deruxtecan) may all lead to ILD at various levels.

**Table 1 Incidences of interstitial lung disease due to different types of anticancer drugs**

| Type of anticancer drug | Incidence of DILD reported in the literature (%) |
|---|---|
| Chemotherapeutic agents[6] | 1.0–40.0 |
| TKIs[7–10] | 0.4–5.3 |
| mTOR inhibitors[11, 12] | 3.0–54.0 |
| ICIs[13–17] | 0.9–3.6 |
| ADCs[18, 19] | 1.9–15.8 |

DILD: drug-induced interstitial lung disease; TKI: tyrosine kinase inhibitor; mTOR: mammalian target of rapamycin; ICI: immune checkpoint inhibitor; ADC: antibody-drug conjugate

## 2 PATHOGENETIC MECHANISMS

The pathogenetic mechanisms of ILD induced by anticancer drugs remain largely unknown, although two possible mechanisms are most recognized: direct cytotoxic effects and immune-mediated injury[20–24]. 1) Cytotoxic drugs may directly damage the type I alveolar epithelial, vascular endothelial, or airway epithelial cells. 2) The drug may act as a hapten or mimic a host antigen to activate immune cells, resulting in a series of immunogenic reactions[20]. These two mechanisms are likely influenced by various host and environmental factors, including age, baseline pulmonary condition before drug administration, and genetic predisposition characterized by the expression of drug metabolism- or immune-related genes, which are ultimately involved in the pathogenesis of DILD.

## 3 CLINICAL MANIFESTATIONS

The disease courses of ILD caused by different types of anticancer drugs vary widely. DILD can occur within a short time after drug administration, from several days to weeks, or it can develop as late as several months after drug administration[25].

DILD lacks specific clinical manifestations. Patients may have no obvious symptoms (usually found by routine chest imaging examination). As their disease progresses, they may develop dry cough and dyspnea on exertion, and some may experience systemic symptoms such as fatigue and fever[26]. Increased respiratory rate and cyanosis might be detected upon physical exam. Pulmonary auscultation is usually normal, but moist or Velcro rales can be heard in some patients[3]. In patients with pre-existing pulmonary diseases, if respiratory symptoms and/or signs worsen during the use of anticancer drugs, DILD should be suspected, and a differential diagnosis should be made between DILD and other pulmonary conditions[3].

## 4 IMAGING AND PATHOLOGICAL FINDINGS

### 4.1 What Are the Common Imaging and Pathological Patterns of DILD?

DILD is not characterized by a specific imaging or pathological pattern. Even so, chest imaging, especially high-resolution computed tomography (HRCT), plays a major role in assessing abnormal pulmonary manifestations during anticancer drug administration. Table 2 summarizes the common imaging and pathological patterns of DILD. Typical DILD radiographic images on HRCT were shown in fig. 1.

### 4.2 When Should a Patient with Suspected DILD Undergo a Chest CT Scan?

For patients using anticancer drugs, close attention should be paid to changes in their respiratory symptoms. If a patient with anticancer drug exposure develops new respiratory symptoms or experiences worsened pre-existing symptoms, a chest CT scan should be performed as early as possible, and HRCT is recommended. HRCT is more sensitive (sensitivity >90%) than conventional chest X-rays and can show interstitial lung changes more clearly. Therefore, it is recommended for early detection of interstitial lung abnormalities[37].

In patients with no obvious respiratory symptoms receiving anticancer drug therapies, adequate attention should be paid if new lung lesions are detected during routine follow-up examinations, and anticancer DILD should always be suspected.

### 4.3 What Is the Recommended Frequency of Chest CT Examination When Using Anticancer Drugs that May Cause DILD?

There is insufficient evidence to support the shortening of chest CT interval when patients are administered anticancer drugs that may cause DILD. Therefore, it is still recommended that a non-contrast CT scan be performed at baseline before administering anticancer drugs and subsequent imaging evaluation be performed with routine tumor monitoring.

Based on routine imaging follow-up, when patients receiving anticancer drugs present with respiratory or mild symptoms, they should be taken seriously, and a chest HRCT scan is recommended as soon as possible.

### 4.4 What Is the Frequency of Subsequent Chest CT follow-up for DILD Patients?

The frequency of CT follow-up in DILD patients depends mainly on the type of DILD lesions. For patients

**Table 2 Common imaging and pathological manifestations of DILD[27–36]**

| Patterns | Chest HRCT manifestations | Pathological manifestations |
|---|---|---|
| DAD (AIP/ARDS)[27–30, 33, 35] | Bilateral extensive ground glass opacity and air-space consolidation in the exudative phase; traction bronchiectasis and reduced lung volume in the fibrotic phase | The hyaline membrane in alveolar space, type II alveolar epithelial hyperplasia, alveolar septum widening, and hyperplasia of loose fibrous tissue. However, observing an acute or exudative phase with abundant hyaline membrane on pathology is difficult. |
| UIP[27–29, 31–34, 36] | Honeycombing and traction bronchiectasis and capillary bronchiectasis, with ground glass opacity and reticular shadows | (1) Dense fibrosis; (2) fibroblastic foci; (3) patchy distribution of lesions in peripheral pulmonary regions and under the pleura, with normal lung tissue observed; (4) presence or absence of pulmonary honeycombing |
| NSIP[27–29, 31–34, 36] | Diffuse distribution in both lungs, mainly in the middle and inferior lobes, with predominantly ground glass opacities and reticular shadows with or without traction bronchiectasis | Homologous distribution of fibroblastic foci; lymphocytes and plasma cells present in the alveolar septum and peribronchial space |
| OP[27–29, 33, 36] | Multifocal patchy consolidation opacities in peribronchovascular regions; the presence of "reversed halo sign" | Polypoid hyperplasia of fibroblast and mucoid stroma in the alveolar space and respiratory bronchiole |
| EP[27–29, 33, 36] | Unilateral or bilateral, nonsegmental consolidation or ground glass opacity; mostly transient changes | Alveolar space filled with a large number of eosinophils and may also contain fibrin and some red blood cells. |
| HP[27–29, 33, 36] | Ground glass opacity in both lung fields, centrilobular nodule with unclear boundary, and Westermark sign | Cell inflammation and loose granulomatous nodules in bronchiole and their peripheral lung tissues |

DAD: diffuse alveolar damage; AIP/ARDS: acute interstitial pneumonia/acute respiratory distress syndrome; UIP: usual interstitial pneumonia; NSIP: nonspecific interstitial pneumonia; OP: organizing pneumonia; EP: eosinophilic pneumonia; HP: hypersensitivity pneumonia

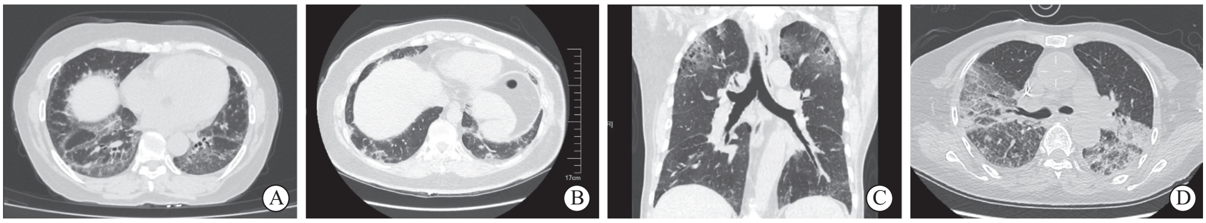

**Fig. 1** Drug-induced interstitial lung disease radiographic images (HRCT)
A: NSIP-like changes; B: OP-like changes; C: HP-like changes; D: DAD-like changes. NSIP, nonspecific interstitial pneumonia; OP, organizing pneumonia; HP, hypersensitivity pneumonia; DAD, diffuse alveolar damage with progressive aggravation of respiratory symptoms, an increased frequency of HRCT examination is recommended. For DILD, mainly present as chronic fibrotic lesions, HRCT can be repeated every 3–6 months[38].

### 4.5 Is Bronchoscopy Required for Patients with Suspected DILD?

For patients with a history of anticancer drug therapy, bronchoscopy is a useful diagnostic tool if clinically feasible. According to the recommendations of the American Thoracic Society/European Respiratory Society (ATS/ERS), although bronchoalveolar lavage fluid (BALF) has no definitive value in the diagnosis of drug-induced lung injury, it can help rule out other etiologies, such as infection, pulmonary alveolar hemorrhage, or tumor, thereby aiding with the differential diagnosis of DILD[39]. Therefore, BALF total cell count and differential counting should be tested whenever available.

### 4.6 Is Lung Biopsy Required for Patients with Suspected DILD?

Whether lung biopsy is required for patients with suspected DILD depends on clinical conditions. Lung biopsy may be needed if the patient's laboratory tests and imaging findings cannot clarify the specific types of lung injury or if distinct treatment strategies are required depending on the differential diagnosis (e.g., drug-induced lung injury and lung infection or tumor progression). Lung biopsy is an important diagnostic tool for highly suspected tumor progression patients. In addition, characteristic pathological features can be revealed and may provide information for subsequent treatments.

## 5 DIAGNOSTIC PROCEDURES AND DIFFERENTIAL DIAGNOSIS

Diagnosing and managing anticancer DILD is a major challenge in clinical practice. Diagnosis is difficult due to various tumors, anticancer drugs, and nonspecific and diverse clinical manifestations. Therefore, multidisciplinary management is particularly important.

During the diagnosis, treatment, and management of DILD, a multidisciplinary team (MDT) of oncologists, pulmonologists, radiologists, pharmacologists, and pathologists can improve the accuracy of diagnosis, especially in patients with inconsistent HRCT and histopathological findings. Multidisciplinary management is also recommended for patients with suspected DILD. In addition, if DILD is highly suspected upon MDT discussion, and treatment with steroids is effective, the diagnosis of DILD will be supported indirectly.

### 5.1 What Are the Timings for Transfer from the Oncology to the Respiratory Department or Consultation from Pneumologists?

MDT is crucial for early detection, accurate diagnosis, clinical management, and prognostic improvement in patients with DILD. If patients develop new symptoms such as tightness in the chest, dyspnea, or cough (particularly a dry cough), or if pre-existing respiratory symptoms worsen, chest imaging shows persistent lung opacity following anticancer drug treatment, recommendations are to conduct a prompt MDT consultation to make management decisions. The diagnostic flowchart of anticancer DILD was summarized in fig. 2.

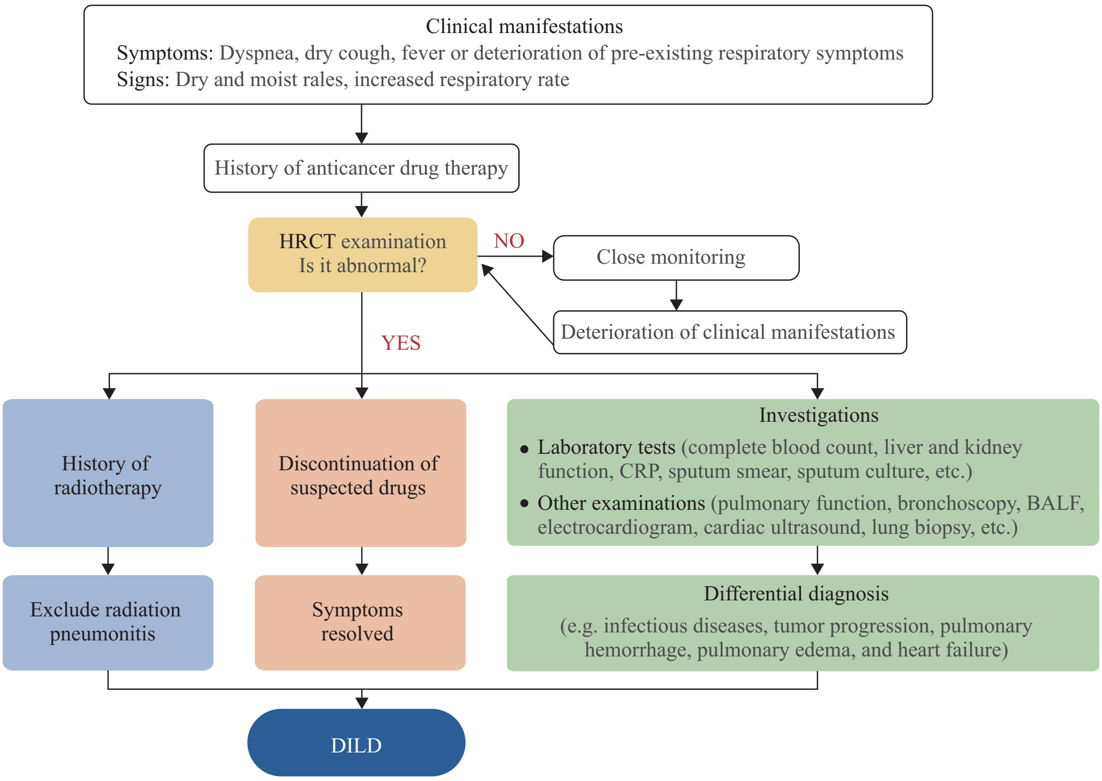

**Fig. 2** Diagnostic flowchart of anticancer DILD

HRCT, high-resolution computed tomography; CRP, C-reactive protein; BALF, bronchoalveolar lavage fluid; DILD, drug-induced interstitial lung disease

### 5.2 Diagnostic Criteria

When diagnosing anticancer DILD, clinicians must remember that many anticancer drugs can cause lung injury at any medication time, even after the treatment is completed. When patients on anticancer therapy develop new lung lesions, we need to consider the possibility of drug-induced lung injury and perform a series of differential diagnoses. The Fleischer Society proposed the following diagnostic criteria after summarizing and amending the previous diagnostic criteria for DILD[27]: (1) newly identified pulmonary parenchymal opacities at CT or chest radiography, commonly in a bilateral nonsegmental distribution; (2) temporal association of presentation with the initiation of a systemic therapeutic agent; and (3) exclusion of other likely causes. The laborotary tests that might be used in diagnosing DILD were summarized in table S1.

### 5.3 Differential Diagnosis

Anticancer drug therapy may cause adverse events in patients, including bone marrow suppression and the impaired function of other organs. In addition, cancer patients may receive other anticancer therapies, such as radiotherapy. Therefore, the differential diagnosis of anticancer DILD is more complicated, including lung infection, tumor progression, lung metastasis, radiation pneumonitis, diffuse alveolar hemorrhage, pulmonary edema, and lung disorders caused by abnormal function in other organs (table 3).

**Table 3 Differential diagnosis for DILD[40–45]**

| Disease | Clinical manifestations | Key diagnostic points |
|---|---|---|
| Infectious diseases | | |
| Bacterial pneumonia | Symptoms vary due to different infectious agents, including fever, cough, dyspnea, purulent sputum, or blood sputum. Tachypnoea and lung consolidation signs may appear on physical examination. | Bacterial pneumonia can be ruled out by laboratory tests such as serologic tests and microbiological culture, and if necessary, a PCR or NGS of BALF can be performed to identify the pathogens. |
| Viral pneumonia | Fever, headache, fatigue, joint pain, and cough. Severe symptoms include tachypnoea, cyanosis, and dry or moist rales in the lungs. | The test of nucleic acid or antigen of respiratory viruses is positive, and the virus serology antibody titer test is positive. |
| Fungal pneumonia | Cough, white foamy mucus sputum, and fever. On physical examination, moist rales can be heard in the lungs, and severe patients may experience tachypnoea. | Fungal pneumonia should be considered when there is a lack of clinical improvement despite broad-spectrum antibiotic therapy. The detection of pathogenic fungi through histopathological or culture methods from sterile sites can diagnose fungal pneumonia provenly. Other tests include serum-specific antibody testing and BALF testing. |
| Tumor progression (e.g., lung cancer, lung metastases, lymphangitis carcinomatosis) | Fever, dyspnea, productive cough, etc. | Elevation of tumor markers might be seen in blood tests, and PET/CT scans could help to identify hypermetabolic lesions. If necessary, a lung biopsy or NGS can be performed. |
| Radiation pneumonitis | Cough, dyspnea after activity, and some patients may have a fever. | A history of radiotherapy, commonly exceeding 30–40 Gy; Typical CT features are GGOs which may increase in density and con-solidate over time. |
| Abnormal function of other organs (e.g., heart failure, pulmonary vascular diseases) | Symptoms are associated with primary diseases, such as edema, cough, orthopnea, pink foamy sputum, and extensive bubbling sounds in both lung fields. | Corresponding tests and examinations should be performed based on medical history and clinical manifestations. |

PCR: polymerase chain reaction; BALF: bronchoalveolar lavage fluid; NGS: next-generation sequencing; PET/CT: positron emission tomography/computed tomography; CT: computed tomography; GGO: ground glass opacity

## 6 TREATMENT

### 6.1 Identification of High-risk Population

Several risk factors for DILD have been identified. Nonspecific risk factors include age (children and those aged >60 years), smoking, history of occupational exposure, presence of lung lesions at baseline (especially interstitial pneumonia), history of pulmonary surgery, decreased respiratory function, history of radiation exposure to the lung, impaired renal function (increased blood concentrations of causative drugs), Eastern Cooperative Oncology Group Performance Status (ECOG) ≥2, and small body surface area[46]. Before starting anticancer treatment, chest imaging and blood tests should be performed to exclude important risk factors. For patients with high-risk factors, anticancer drugs with lower risks of pulmonary toxicity should be selected within a reasonable range based on careful consideration and benefit-risk weighing; respiratory symptoms and signs of patients should be closely monitored during administration.

### 6.2 What Are the Precautions during Administering Anticancer Drugs to Avoid DILD or Identify DILD as Early as Possible?

Combination therapy featuring anticancer drugs may increase the incidence of pulmonary toxicity. A previous study reported that the incidence of DILD was 2.1% when erlotinib was combined with gemcitabine to treat pancreatic cancer; this incidence was higher than that for gemcitabine alone[47]. Another study has reported an incidence of pulmonary toxicity of approximately 1.4% for gemcitabine monotherapy and up to 33% when combined with paclitaxel[25]. The combination of PD-1 or PD-L1 inhibitors with CTLA-4 inhibitors can significantly increase the incidence of pneumonitis, with an incidence as high as 10%[48]. DILD induced by combination therapy is often more severe and needs a longer recovery than monotherapy. In clinical practice, the combination of drugs with a high risk of pulmonary toxicity, such as ICIs combined with small molecular targeting agents, should be avoided if possible[49]. Furthermore, studies have shown that combination with radiotherapy may also lead to an increased risk of pulmonary toxicity. For example, the incidence of radiation pneumonitis can be as high as 29% in patients treated with anastrozole and paclitaxel in combination with radiotherapy[50]. In addition, the package insert for gemcitabine clearly specifies that the optimum regimen for safe administration of gemcitabine with therapeutic doses of radiation has not been determined in all tumor types (due to the risk of severe pneumonitis and esophagitis when given concurrently with radiotherapy)[51]. Therefore, when radiotherapy is combined with chemotherapy or targeted agents, patients should be closely monitored for pulmonary symptoms and signs, and HRCT should be performed promptly if necessary[25].

### 6.3 Grading of DILD

Oncologists generally accept the following two grading criteria in clinical practice: (1) the grading of pulmonary toxicity/pneumonitis by the American Society of Clinical Oncology (ASCO) guideline on the management of immune-related adverse events (table 4), and (2) the grading of pneumonitis according to the National Cancer Institute-Common Terminology Criteria for Adverse Events version 5.0 (NCI-CTCAE v5.0) (table 5). In both grading methods, pulmonary toxicity/pneumonitis is defined as the presence of focal or diffuse inflammation of the lung parenchyma. The former grading method focuses primarily on the degree of lung lobes affected by DILD, while the latter is easier to apply clinically[2].

**Table 4 Grading of pulmonary toxicity in ASCO guideline on the management of immune-related adverse events[52]**

| Grade | Description |
|---|---|
| G1 | Asymptomatic; confined to one lobe of the lung or 25% of the lung parenchyma; clinical or diagnostic observations only |
| G2 | Symptomatic; involving more than one lobe of the lung or 25%–50% of lung parenchyma; medical intervention indicated; limiting instrumental ADL |
| G3 | Severe symptoms; hospitalization required; involving all lung lobes or >50% of lung parenchyma; limiting self-care ADL; oxygen indicated |
| G4 | Life-threatening respiratory compromise; urgent intervention indicated (tracheotomy or intubation) |

ASCO: American Society of Clinical Oncology; ADL: activity of daily living

**Table 5 Grading of pneumonitis according to CTCAE v5.0[53]**

| Grade | Description |
|---|---|
| G1 | Asymptomatic; clinical or diagnostic observations only; intervention not indicated |
| G2 | Symptomatic; intervention indicated; limiting instrumental ADL |
| G3 | Severe symptoms; limiting self-care ADL, oxygen indicated |
| G4 | Life-threatening respiratory compromise; urgent intervention indicated (e.g., tracheotomy or intubation) |
| G5 | Death |

CTCAE: common terminology criteria for adverse events; ADL: activity of daily living

### 6.4 Principles for the Grading Management of DILD

The general principle for the grading management of DILD is that asymptomatic patients (i.e., grade 1) usually do not need drug withdrawal but should be closely monitored. If the condition worsens or there are new symptoms (i.e., grade 2), anticancer drugs should be discontinued immediately, and steroids should be administered. If symptoms are severe or life-threatening (grades 3–4), anticancer drugs should be discontinued permanently, steroid treatment should be given, and if necessary, other therapies, including immunosuppressive agents, can be considered. DILD management strategies vary slightly among different types of anticancer drugs[54–57] (table 6).

### 6.5 How to Use Steroids Rationally?

Glucocorticoids are commonly used in DILD patients to improve symptoms and promote the repair of lung injury, especially in moderate and severe cases of DILD and in the acute onset of DILD[44, 58]. However, there are currently no results from large-scale studies regarding the dose and duration of steroids used in treating DILD. Furthermore, no clinical trials have been conducted to demonstrate the efficacy of corticosteroids in DILD patients.

In clinical practice, it is recommended that the management of DILD refers to the grade of severity. Individualized treatment strategies should consider the patients' pre-existing diseases, the type of neoplasm, the severity of other adverse reactions, and steroid tolerance to reduce the risk of potential complications. Pneumologists and endocrinologists' consultations or MDT consultations may be considered if necessary.

### 6.6 What Other Treatments Can Be Attempted besides Steroids?

Alternative drugs may be selected for treatment if patients are insensitive to steroids or if the use of steroids is limited due to the history of other underlying diseases:

(1) Immunosuppressive agents: For patients with ICI-induced ILD and symptoms not resolved 48 h after steroid therapy, it is recommended to administer immunosuppressive agents, such as infliximab, mycophenolate mofetil or intravenous immunoglobulins[59, 60]. However, it should be noted that the evidence for treating immune-related adverse events with immunosuppressive therapy mainly arises from immune-related colitis. Such treatment for immune-related DILD needs further investigation.

(2) Antagonists: If the metabolic period of a drug is long or there are many residual components, antagonists may be considered to inhibit or relieve lung injury. For example, neostigmine is used to antagonize respiratory depression induced by aminoglycoside antibiotics; calcium is used to antagonize polymyxin-induced respiratory failure.

(3) Supportive care, including oxygen therapy,

**Table 6 Management of ILD induced by different anticancer drugs***

| | G1 | G2 | ≥G3 |
|---|---|---|---|
| TKI[54] | Proceed with close monitoring. If it worsens, treat it as G2 or ≥G3. | Hold TKIs until clinical improvement to ≤G1.<br>Prednisolone at a starting dose of 0.5–1 mg/kg/day or equivalent corticosteroids for 2–4 weeks, and taper over 6 weeks after symptoms and signs are relieved.<br>Chest CT±bronchoscopy and BALF;<br>Consider oxygen therapy | Permanent discontinuation of TKIs is recommended.<br>G4: methylprednisolone 500–1000 mg/day for 3 days for pulse therapy, then prednisone 1–2 mg/kg/day or equivalent for 2–4 weeks and taper after signs and symptoms relieved. The total course of treatment should be at least 8 weeks.<br>Chest CT±bronchoscopy and BALF.<br>Empiric antibiotics may be considered.<br>Oxygen therapy±mechanical ventilation if necessary |
| ICI[49] | Hold ICIs or proceed with close monitoring.<br>Repeat chest CT in 3–4 weeks. Consider resuming treatment in case of radiographic improvement.<br>If there is no improvement, treat it as G2. | Hold ICIs until clinical improvement to ≤G1.<br>Methylprednisolone (Ⅳ) 1–2 mg/kg/day or equivalent). If symptoms improve after 48–72 h, taper the doses of steroids at 5–10 mg/week over 4–6 weeks; if there is no improvement, treat it as ≥G3; If the infection cannot be completely ruled out, consider empiric antibiotics.<br>Repeat chest CT in 3–4 weeks.<br>If improvement to ≤G1, consider ICIs rechallenge after careful evaluation. | Permanently discontinue ICIs and consider hospitalization.<br>Empiric antibiotics are recommended if the infection is not completely ruled out.<br>Pulmonary and infectious disease consults if necessary.<br>Methylprednisolone (Ⅳ) 2 mg/kg/day or equivalent, consider pulmonary ventilation if appropriate; if clinical symptoms improve after 48 h, continue treatment until improvement to ≤G1 and then taper over 4–6 weeks; if no significant improvement, consider Ⅳ of infliximab (5 mg/kg) (may repeat after 14 days), or mycophenolate mofetil 1–1.5 g/dose, 2 times/day, or immunoglobulin |
| ADC△[18, 19] | Hold ADCs until full recovery.<br>Resume treatment if resolved in ≤28 days after onset; reduce dose one level if resolved in >28 days; discontinue treatment if it occurs after day 22 and is not resolved within 49 days after the last infusion.<br>Consider follow-up imaging in 1–2 weeks (or clinically indicated).<br>Consider starting steroids (e.g., ≥0.5 mg/kg/day prednisone or equivalent) until improvement, and then taper over ≥4 weeks.<br>If deterioration after steroids therapy, treat it as G2. | Permanently discontinue.<br>Immediately start steroids (e.g., ≥1 mg/kg/day prednisone or equivalent) for ≥14 days until complete resolution of clinical and chest CT findings, and then taper over at least ≥4 weeks.<br>Close monitoring of symptoms;<br>Repeat chest imaging as clinically indicated.<br>If clinical or diagnostic findings worsen or do not improve within 5 days: Consider increasing the dose of steroids (e.g., 2 mg/kg/day prednisone or equivalent) or switch to intravenous administration (e.g., methylpred-nisolone).<br>-Reconsider additional follow-up for other etiologies.<br>-Escalate care if clinically indicated | Permanently discontinue.<br>Hospitalization indicated.<br>Immediately start empiric high-dose methylprednisolone Ⅳ (e.g., 500–1000 mg/day for 3 days), followed by prednisone ≥1 mg/kg/day (or equivalent) for ≥14 days or until complete resolution of clinical and chest CT findings, and then taper over ≥4 weeks.<br>Repeat chest CT if clinically indicated.<br>If there is no improvement in 3–5 days,<br>-Reconsider additional follow-up for other etiologies;<br>-Consider other immunosuppressive agents and/or treat them according to local practice |

*This is a general management principle for ILD induced by different anticancer drugs, the management may vary between specific drugs, and it is recommended to refer to the package insert. △For ADC-induced DILD, clinical management guidance of T-DXd-related ILD is referred. TKI, tyrosine kinase inhibitors; CT, computed tomography; BALF, bronchoalveolar lavage fluid; ICI: immune checkpoint inhibitors; ILD: intestitial lung disease; ADC: antibody-drug conjugate mechanical ventilation, fluid therapy, sedation, and spasmolysis.

(4) Antifibrotic therapy: Fibrosis is an important manifestation of chronic DILD. Commonly used antifibrotic agents in clinical practice include nintedanib[61] and pirfenidone[62]. These drugs can delay the decline of pulmonary function in patients with idiopathic pulmonary fibrosis, significantly reduce the risk of acute exacerbation and hospitalization, and improve impaired pulmonary function[63].

(5) Treatment of pre-existing disease: The pre-existing disease should be actively treated for patients with the underlying disease to reduce the risk for DILD[64].

(6) Traditional Chinese herbal medicine: Clinical studies of small sample size and animal experiments have shown that some single Chinese herbs (e.g., miltiorrhizae and tetrandrine) and combined medications (e.g., feitong oral liquid and feixiankang) might be used for the treatment of ILD. In addition, Chinese traditional medicine also showed some effectiveness in prolonging survival, improving the quality of life, and relieving clinical symptoms[65]. The flow chart of management of anticancer DILD is summarized in fig. 3.

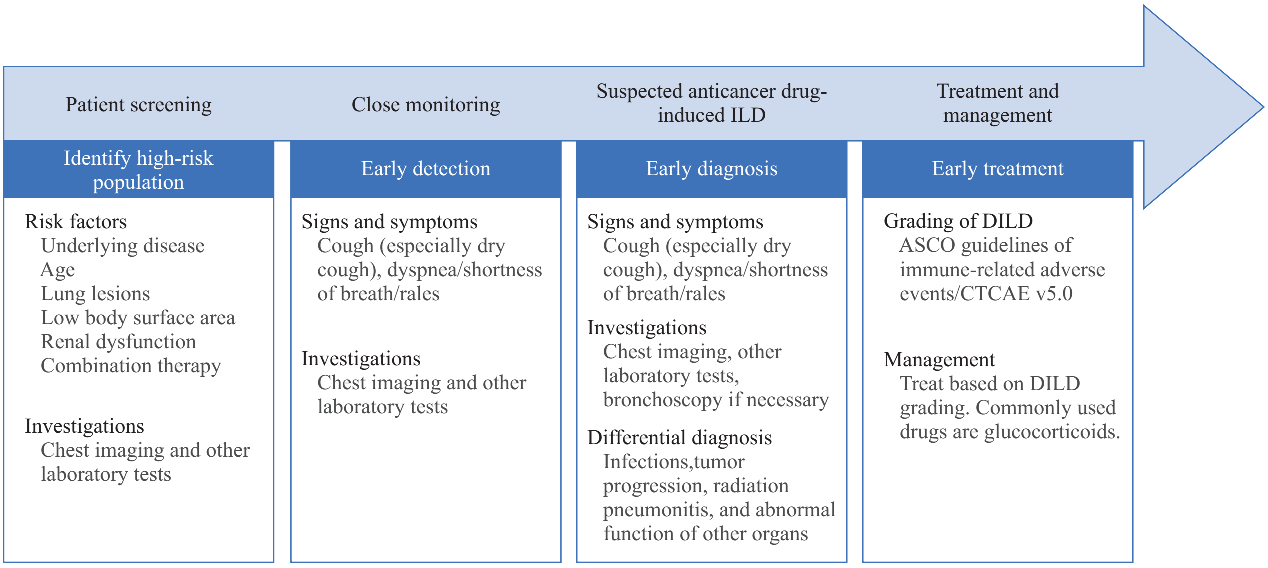

**Fig. 3** Flow chart of management of anticancer DILD

### 6.7 How Do We Rehabilitate Pulmonary Function in DILD Patients after Treatment?

ATS/ERS proposes a comprehensive intervention plan for pulmonary function rehabilitation: (1) Endurance training: Cycling or walking is the most common form of endurance training in pulmonary rehabilitation and is recommended in the rehabilitation plan. The initial training intensity is usually set at 70%–85% of the maximum exercise capacity; (2) Intermittent training: a modification of endurance training in which high-intensity exercise is alternated with periodic rest or low-intensity exercise. Such training is suitable for patients with lung injury who have difficulty achieving target intensities or durations due to dyspnea, fatigue, or other symptoms; (3) Other methods: education, nutritional support, and psychological support are generally included. Oxygen saturation should be properly monitored during exercise. Oxygen therapy should be administered to patients whose oxygen saturation ($SpO_2$) is lower than 85% during training to maintain the $SpO_2$ >88%[66].

### 6.8 Prognosis

The prognosis of patients with anticancer DILD varies by drug and individual differences. Overall, most patients with mild and moderate DILD have a good prognosis. More than two-thirds of patients with ICIs-induced DILD can be relieved or cured by discontinuation of ICIs and treatment with steroids. Patients who are insensitive to steroids have a poor prognosis[37]. TKI-induced ILD usually presents with an earlier onset but is generally milder and responds well to steroids[7–10]. ADC-induced ILD generally occurs around 6 months during treatment, with some ≥grade 3 cases reported but is overall manageable[18, 19].

## 7 SUMMARY

With the rapid development of novel anticancer agents, the incidence of anticancer DILD is expected to increase in the coming years. This consensus aims to raise awareness and improve the understanding of DILD among clinicians, especially oncologists. In clinical practice, early identification of high-risk populations, accurate diagnosis, prompt management, and close monitoring will all contribute to the treatment of DILD and thus improve the prognosis of patients. Establishing a procedural management pattern based on multidisciplinary collaboration is essential. Further research is needed to identify risk factors and understand the mechanisms of DILD.

## 8 SUPPLEMENTARY DATA

**Table S1 Overview of laboratory tests**

| Laboratory tests | Brief introduction |
|---|---|
| Blood tests | Complete blood count, liver function, ESR, CRP, LDH, KL-6, and markers of allergic reaction. DLST can also be used to diagnose DILD or suspected DILD, but false positive or negative reactions may occur. |
| Arterial blood gas | Arterial blood gas usually includes pH, $PaO_2$, $PaCO_2$, $HCO_3^-$, and BE, which are reliable indicators to determine whether there is an acid-base imbalance and hypoxia and the degree of hypoxia. |
| Pulmonary function tests | Pulmonary function tests usually include tidal volume, forced vital capacity, 1-second forced vital capacity, residual volume, and diffusing capacity of the lungs for carbon monoxide. Pulmonary function may be normal in mild cases, but vital and diffusing capacities may decrease significantly in severe cases of interstitial lung disease. |

DILD, drug-induced interstitial lung disease; ESR, erythrocyte sedimentation rate; CRP, C-reactive protein; LDH, lactate dehydrogenase; KL-6, Krebs von den lungen-6; DLST, drug lymphocyte stimulation test; BE, base excess

**Open Access**

This article is licensed under a Creative Commons Attribution 4.0 International License https://creativecommons.org/licenses/by/4.0/), which permits use, sharing, adaptation, distribution and reproduction in any medium or format, as long as you give appropriate credit to the original author(s) and the source, provide a link to the Creative Commons licence, and indicate if changes were made. The images or other third party materials in this article are included in the article's Creative Commons licence, unless indicated otherwise in a credit line to the material. If material is not included in the article's Creative Commons licence and your intended use is not permitted by statutory regulation or exceeds the permitted use, you will need to obtain permission directly from the copyright holder. To view a copy of this licence, visit http://creativecommons.org/licenses/by/4.0/.

**Conflict of Interest Statement**

The authors declare that they have no competing interests.

Author Hua-ping DAI is a member of the Editorial Board for Current Medical Science. The paper was handled by other editors and has undergone rigorous peer review process. Author Hua-ping DAI was not involved in the journal's review of, or decisions related to, this manuscript.

**Appendix**

Expert Consensus Panel of Anticancer Drug-related Interstitial Lung Disease Diagnosis and Treatment Group

**Consultants:**

Chen WANG, National Respiratory Center, National Clinical Research Center for Respiratory Disease, Institute of Respiratory Medicine, Chinese Academy of Medical Sciences, Respiratory Center of China-Japan Friendship Hospital;

Bing-he XU, National Cancer Center, National Clinical Research Center for Cancer, Cancer Hospital Chinese Academy of Medical Sciences and Peking Union Medical College

**Authors:**

Hua-ping DAI, Department of Pulmonary and Critical Care Medicine, China-Japan Friendship Hospital, National Center for Respiratory Medicine, Chinese Academy of Medical Sciences;

Fei MA, Department of Medical Oncology, National Cancer Center, National Clinical Research Center for Cancer, Cancer Hospital Chinese Academy of Medical Sciences and Peking Union Medical College;

Yan-hong REN, Department of Pulmonary and Critical Care Medicine, China-Japan Friendship Hospital, National Center for Respiratory Medicine, Chinese Academy of Medical Sciences;

Shan-shan CHEN, Department of Medical Oncology, National Cancer Center, National Clinical Research Center for Cancer, Cancer Hospital Chinese Academy of Medical Sciences and Peking Union Medical College;

Yi-qun LI, Department of Medical Oncology, National Cancer Center, National Clinical Research Center for Cancer, Cancer Hospital Chinese Academy of Medical Sciences and Peking Union Medical College

**Members (in alphabetical order by last name):**

Qi-hang CHEN, Department of Radiology, Beijing Hospital;

Shan-shan CHEN, Department of Medical Oncology, National Cancer Center, National Clinical Research Center for Cancer, Cancer Hospital Chinese Academy of Medical Sciences;

Jiu-wei CUI, Department of Medical Oncology, Bethune First Hospital of Jilin University;

Hua-ping DAI, Department of Pulmonary and Critical Care Medicine, China-Japan Friendship Hospital, National Center for Respiratory Medicine, Chinese Academy of Medical Sciences;

Ji-feng FENG, Department of Medical Oncology, Jiangsu Cancer Hospital;

Rui-e FENG, Department of Pathology, Peking Union Medical College Hospital;

Jun GUO, Department of Renal Cancer and Melanoma, Beijing Cancer Hospital;

Xi-chun HU, Department of Medical Oncology, Fudan University Shanghai Cancer Center;

Hong-bing HUANG, Department of Pharmacy, Sun Yat-sen University Cancer Center;

Han-dong JIANG, Department of Respiration, Renji Hospital, Shanghai Jiaotong University School of Medicine;

Zheng-yu JIN, Radiology Department, Peking Union Medical College Hospital;

Guo-hui LI, Department of Medical Oncology, National Cancer Center, National Clinical Research Center for Cancer, Cancer Hospital Chinese Academy of Medical Sciences;

Hui-ping LI, Department of Respiratory Medicine, Shanghai Pulmonary Hospital, Tongji University;

Jing LI, Department of Radiology, National Cancer Center, National Clinical Research Center for Cancer, Cancer Hospital Chinese Academy of Medical Sciences;

Yi-qun LI, Department of Medical Oncology, National Cancer Center, National Clinical Research Center for Cancer, Cancer Hospital Chinese Academy of Medical Sciences;

Hong-yuan LI, Department of Breast and Thyroid Surgery, the First Affiliated Hospital of Chongqing Medical University;

Tian-shu LIU, Department of Medical Oncology, Fudan University Zhongshan Hospital;

Qun LUO, Department of Respiratory Medicine, the First Affiliated Hospital of Guangzhou Medical University;

Fei MA, Department of Medical Oncology, National Cancer Center, National Clinical Research Center for Cancer, Cancer Hospital Chinese Academy of Medical Sciences;

Jie MENG, Department of Pulmonary and Critical Care Medicine, the Third Xiangya Hospital of Central South University;

Qu-chang OUYANG, Department of Medical Oncology, Hunan Cancer Hospital;

Yue-yin PAN, Department of Medical Oncology, Anhui Provincial Hospital;

Yan-hong REN, Department of Pulmonary and Critical Care Medicine, China-Japan Friendship Hospital, National Center for Respiratory Medicine, Chinese Academy of Medical Sciences;

Lin SHEN, Department of Gastrointestinal Oncology, Beijing Cancer Hospital;

Ye-hui SHI, Department of Medical Oncology, Tianjin Medical University Cancer Institute and Hospital;

Lu SI, Department of Renal Cancer and Melanoma, Beijing Cancer Hospital;

Tao SUN, Department of Medical Oncology, Liaoning

Cancer Hospital and Institute;
Tong SUN, Pathology Department, Yale New Haven Health/ Bridgeport Hospital;
Da-bei TANG, Department of Medical Oncology, Harbin Medical University Cancer Hospital;
Yue'e TENG, Department of Medical Oncology, The First Hospital of China Medial University;
Zhong-sheng TONG, Department of Medical Oncology, Tianjin Medical University Cancer Institute and Hospital;
Jian-wei WANG, Department of Radiology, National Cancer Center, National Clinical Research Center for Cancer, Cancer Hospital Chinese Academy of Medical Sciences;
Shu-lian WANG, Department of Radiation Therapy, National Cancer Center, National Clinical Research Center for Cancer, Cancer Hospital Chinese Academy of Medical Sciences;
Shu-sen WANG, Department of Medical Oncology, Sun Yat-sen University Cancer Center;
Yu YAO, Department of Medical Oncology, the First Affiliated Hospital of Xi'an Jiaotong University;
HongYAN, Department of Medical Oncology, Peking Union Medical College Hospital;
Jian-ming YING, Department of Pathology, National Cancer Center, National Clinical Research Center for Cancer, Cancer Hospital Chinese Academy of Medical Sciences;
Qing ZHAI, Pharmacy Department, Fudan University Shanghai Cancer Center;
Hui-lan ZHANG, Department of Respiratory Medicine, Tongji Hospital, Tongji Medical College, Huazhong University of Science and Technology;
Jian ZHANG, Department of Medical Oncology, Fudan University Shanghai Cancer Center;
Qing-yuan ZHANG, Department of Medical Oncology, Harbin Medical University Cancer Hospital;
Xiao-tian ZHANG, Department of Gastrointestinal Oncology, Beijing Cancer Hospital;
Yong-qiang ZHANG, Department of Medical Oncology, Beijing Hospital;
Wei-hong ZHAO, Department of Medical Oncology, First Center of Medicine, Chinese PLA General Hospital;
Ai-ping ZHOU, Department of Medical Oncology, National Cancer Center, National Clinical Research Center for Cancer, Cancer Hospital Chinese Academy of Medical Sciences;
Cai-cun ZHOU, Department of Medical Oncology, Shanghai Pulmonary Hospital, Tongji University;
Cheng-zhi ZHOU, Department of Respiratory Medicine, the First Affiliated Hospital of Guangzhou Medical University;
Jian-ying ZHOU, Department of Respiratory Medicine, the First Affiliated Hospital, Zhejiang University;
Jun ZHU, Department of Lymphoma and Hematologic Oncology, Beijing Cancer Hospital
